# Draft Genome Sequence of *Lactobacillus delbrueckii* subsp. *bulgaricus* CFL1, a Lactic Acid Bacterium Isolated from French Handcrafted Fermented Milk

**DOI:** 10.1128/genomeA.00052-16

**Published:** 2016-03-03

**Authors:** Julie Meneghel, Eric Dugat-Bony, Françoise Irlinger, Valentin Loux, Marie Vidal, Stéphanie Passot, Catherine Béal, Séverine Layec, Fernanda Fonseca

**Affiliations:** a UMR GMPA, AgroParisTech, INRA, Université Paris-Saclay, Thiverval-Grignon, France; b MaIAGE, INRA, Université Paris-Saclay, Jouy-en-Josas, France; c INRA, GeT-PlaGe, Genotoul, Castanet-Tolosan, France; d INRA, UAR1209, Castanet-Tolosan, France

## Abstract

*Lactobacillus delbrueckii* subsp. *bulgaricus* (*L. bulgaricus*) is a lactic acid bacterium widely used for the production of yogurt and cheeses. Here, we report the genome sequence of *L. bulgaricus* CFL1 to improve our knowledge on its stress-induced damages following production and end-use processes.

## GENOME ANNOUNCEMENT

*Lactobacillus delbrueckii* subsp. *bulgaricus* (*L. bulgaricus*) is a lactic acid bacterium that is traditionally used as a dairy starter. *L. bulgaricus* is mainly involved in milk acidification and more generally plays a role in the preservation and development of flavor, texture, and vitamins of dairy products (1, 2). In addition, this bacterium is exploited to produce lactic acid for use in the food, cosmetics, and chemical sectors (3).

Aimed at enhancing its industrial utilization, considerable research efforts have been undertaken to characterize the diversity of *L. bulgaricus* strains. Since 2006, the genome sequences of six different strains were published: ATCC 11842 (1), ATCC BAA-365 (4), 2038 (5), CNCM I-1519 and CNCM I-1632 (6), and CRL871 (7). Actually, the production of lactic acid bacteria by traditional fermentation, their stabilization (by freezing and/or drying methods), and final use (in yogurt, cheese-making, or biomolecule production) are responsible for the undesirable degradation of their viability and functionality. Therefore, scientific work has also focused on optimizing the formulation and operating conditions of starter production and the study of *L. bulgaricus* behavior under related stress conditions, especially considering the sensitive strain CFL1 (8–16). However, the majority of these studies are descriptive, and there is a clear need for comprehension of the underlying molecular cellular damage mechanisms.

Consequently, we sequenced the genome of *L. bulgaricus* CFL1 (available at CIRM-BIA, Rennes, France), isolated >20 years ago from handcrafted fermented milk, by using Illumina MiSeq technology. Paired-end sequences (2 × 250-bp long) were merged using FLASH (17) and assembled using SPAdes (version 3.1.1, with default parameters) (18), which generated 44 large contigs (>1,000 bp), with an average sequencing coverage of 115-fold, *N*_50_ contig of 116 Kbp, and a largest contig of 266 Kbp. The unclosed draft genome is 1,757,917 bp in length and has a G+C content of 49.8%. Gene prediction and annotation were performed using the Integrated Microbial Genomes (IMG) system (19), according to the standard operating procedure of the Department of Energy-Joint Genome Institute (DOE-JGI) microbial genome annotation pipeline (MGAP version 4) (20). The genome is composed of 1,882 predicted genes comprising 1,794 coding DNA sequences, 20 rRNAs, and 68 tRNAs.

The analysis of the genome of *L. bulgaricus* CFL1 revealed general consistency with previously sequenced strains, including G+C content, carbohydrate metabolism, and genes involved in exopolysaccharide and lipopolysaccharide production. However, a disparity regarding the detected clustered regularly interspaced short palindromic repeat (CRISPR)-associated (Cas) systems should be emphasized. They consist of defense systems against plasmid and phage invasions that are classified into three major groups (types I, II, and III) (21). Whereas in *L. bulgaricus*, only type II or III seems to be present, if any (22), interestingly, CFL1 appeared to possess both of these CRISPR-Cas system types simultaneously.

The availability of the genome sequence and annotation of *L. bulgaricus* CFL1 will allow a deeper insight into its stress response mechanisms, thus making it possible to identify potential preservation issues and to better understand cell damages following production and end-use processes, especially through transcriptomic and proteomic approaches.

### Nucleotide sequence accession numbers.

The draft genome sequences of *L. bulgaricus* CFL1 have been deposited at the EMBL database under accession numbers CZPS01000001 to CZPS01000044.
